# Supplementary figures and images for: Experimental evolution of Pseudomonas aeruginosa to colistin in spatially confined microdroplets identifies evolutionary trajectories consistent with adaptation in microaerobic lung environments
Source: mBio. 2023 Oct 17;14(6):e01506-23. doi: 10.1128/mbio.01506-23 (PMC10746239; doi:10.1128/mbio.01506-23)

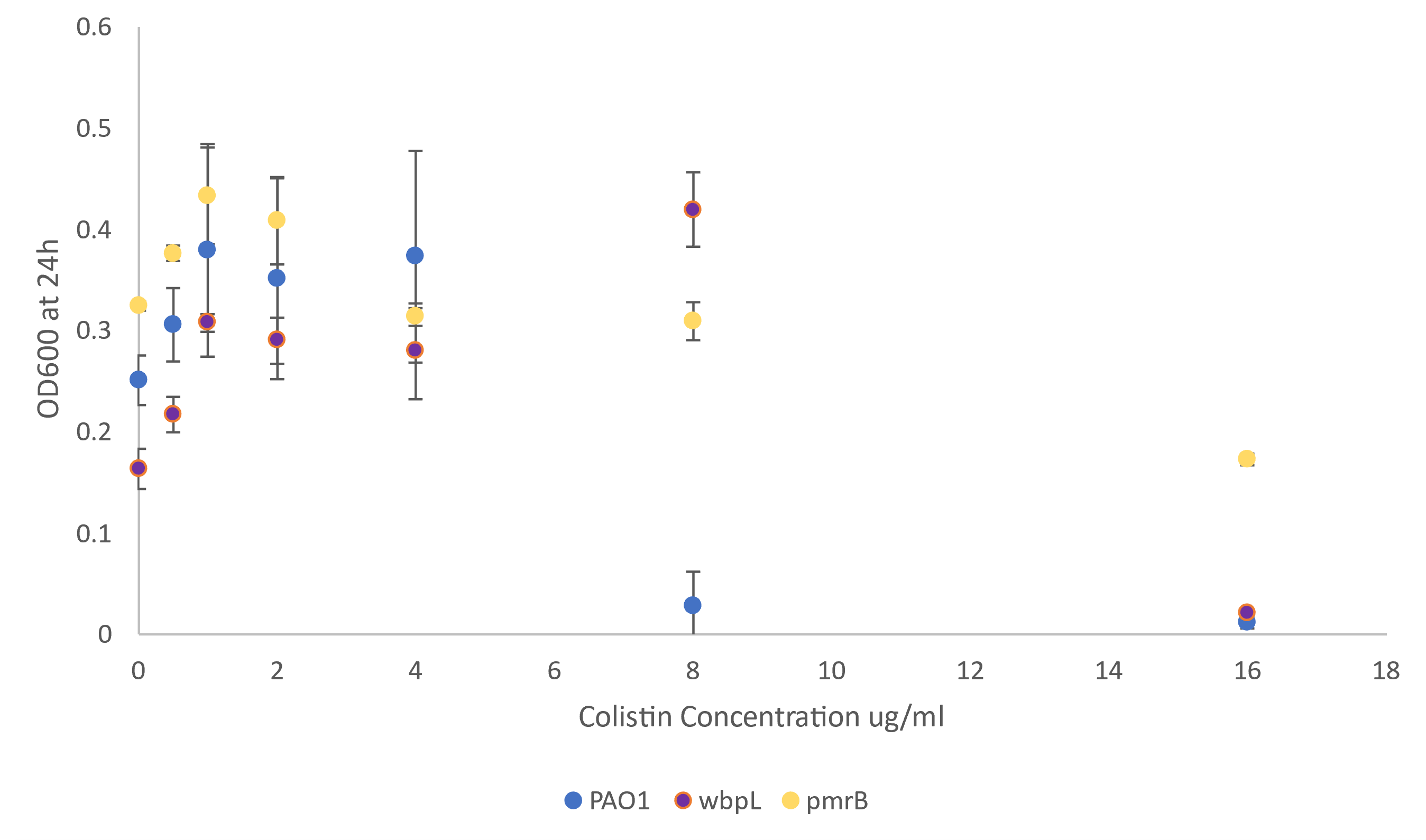

Supplement: Figure S1 — Colistin and growth. [file mbio.01506-23-s0001.tif]

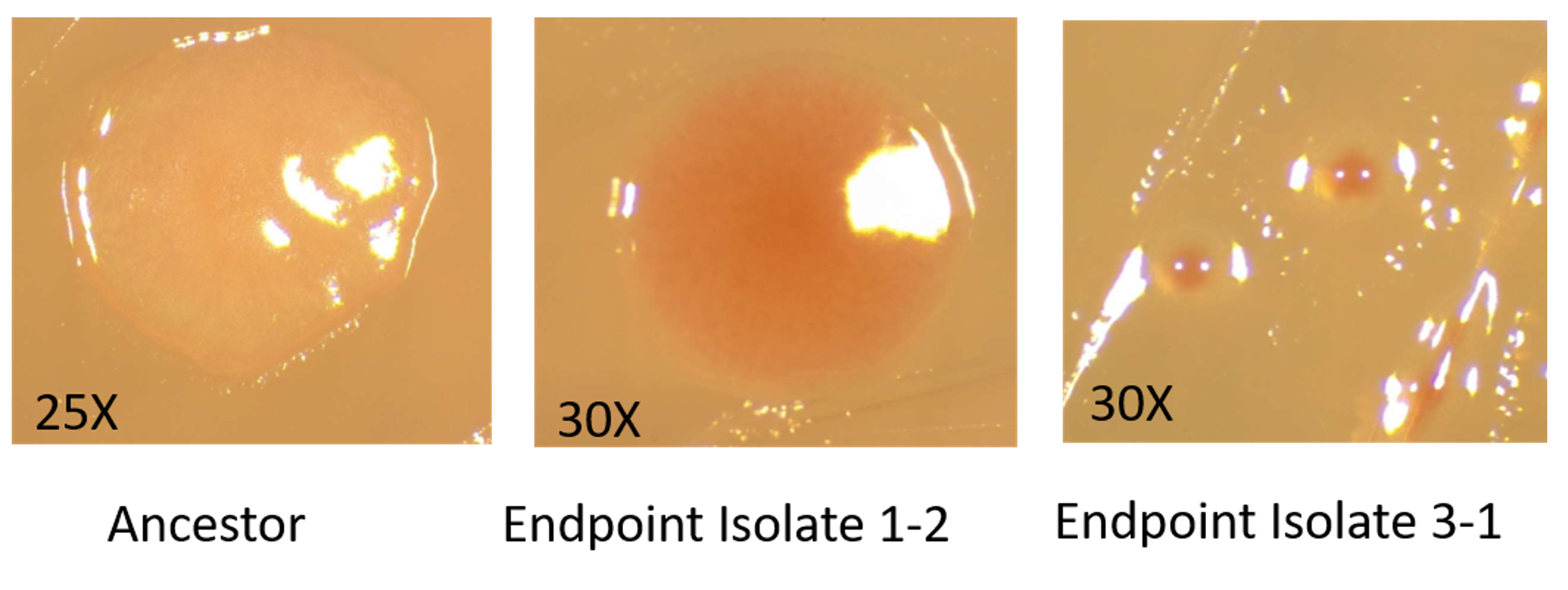

Supplement: Figure S2 — Colony morphologies. [file mbio.01506-23-s0002.tif]
