# Supplementary material for: Experimental evolution of Pseudomonas aeruginosa to colistin in spatially confined microdroplets identifies evolutionary trajectories consistent with adaptation in microaerobic lung environments
Source: mBio. 2023 Oct 17;14(6):e01506-23. doi: 10.1128/mbio.01506-23 (PMC10746239; doi:10.1128/mbio.01506-23)
Supplement: Movie Legend — Legend for the supplemental movie. [file mbio.01506-23-s0003.docx]

**Supporting Information S6**

**“Experimental evolution of *Pseudomonas aeruginosa* to colistin in spatially confined microdroplets identifies evolutionary trajectories consistent with adaptation in microaerobic lung environments”**

Saoirse Disney-McKeethen^a^, Seokju Seo^a^, Heer Mehta^a^, Karukriti Ghosh^a^, and Yousif Shamoo^a*^

^a^Department of BioSciences, Rice University, Houston, Texas, 77005, United States

^*^Correspondence and requests for materials should be addressed to Y.S. (email: shamoo@rice.edu)

**Legend for Supplemental Movie S5**

Videos showing the motility within droplets of the PAO1 Ancestor, EP1-2, EP1-3, *wbpL*^(Δ229)^*,* and *pmrB^(L108Q)^* are accessible in the folder microdroplet_videos as (**S5**). Populations were imaged and recorded inside microdroplets after 24 hours of incubation to examine the spatial distribution and motility of the bacteria within the microdroplets. Images and recordings show that, as expected, the ancestor PAO1 strain is both planktonic and motile; bacteria within microdroplets are moving quickly and are evenly distributed throughout the microdroplets.
