# Supplementary material for: Experimental evolution of Pseudomonas aeruginosa to colistin in spatially confined microdroplets identifies evolutionary trajectories consistent with adaptation in microaerobic lung environments
Source: mBio. 2023 Oct 17;14(6):e01506-23. doi: 10.1128/mbio.01506-23 (PMC10746239; doi:10.1128/mbio.01506-23)
Supplement: Supplemental Legends — Legends For Fig. S1 and S2 and Tables S1 to S4. [file mbio.01506-23-s0004.docx]

**Supplemental Text File**

**“Experimental evolution of *Pseudomonas aeruginosa* to colistin in spatially confined microdroplets identifies evolutionary trajectories consistent with adaptation in microaerobic lung environments”**

Saoirse Disney-McKeethen^a^, Seokju Seo^a^, Heer Mehta^a^, Karukriti Ghosh^a^, and Yousif Shamoo^a*^

^a^Department of BioSciences, Rice University, Houston, Texas, 77005, United States

^*^Correspondence and requests for materials should be addressed to Y.S. (email: shamoo@rice.edu)

**Supplementary Figure 1** Broth microdilution test of growth of PAO1, *wbpL^(Δ229)^*, and *pmrB^(L108Q)^* at 0, 0.5, 1, 2, 4, 6, 8, and 16 μg/ml colistin shows that mutant *wbpL* grows at twofold higher colistin concentration than the ancestor PAO1 strain. Graph shows averaged final OD after 24 hours of continuous growth for each strain at varying colistin concentrations.

**Supplementary Figure 2** shows differing colony morphologies of the PAO1 Ancestor, EP 1-2 (population containing high-frequency mutations *phoQ*, *fleQ,* and *wbpL* mutations), and EP3-1 (population containing high frequency *phoQ* and *fliC* mutations). Typically, increased absorption of Congo Red dye indicates increased exopolysaccharide production.

**SI 1 Colistin Concentration and OD by Day** Colistin gradient and Optical density by Day for each experimental evolution condition

**SI 2** **Growth Curve Data pmrB mutant vs Ancestor Growth Curve** Data for: P. aeruginosa PAO1 pmrB(L108Q) and Ancestor

**SI 3 MIC Broth Dilution** pmrB(L108Q), wbpL(Δ229) and ancestor

**SI 4 Longitudinal Short Read Sequencing Data for all** Allelic frequencies >5% for all experimental populations

**SI 5 Motility in Microdroplets** Visualization of motility and clumping phenotypes in microdroplets. Videos showing the motility within droplets of the PAO1 Ancestor, EP1-2, EP1-3, *wbpL*^(Δ229)^*,* and *pmrB^(L108Q)^* are accessible in the folder microdroplet_videos as (**S5**). Populations were imaged and recorded inside microdroplets after 24 hours of incubation to examine the spatial distribution and motility of the bacteria within the microdroplets. Images and recordings show that, as expected, the ancestor PAO1 strain is both planktonic and motile; bacteria within microdroplets are moving quickly and are evenly distributed throughout the microdroplets.
